# Supplementary material for: Biphasic α2β1 Integrin Expression in Breast Cancer Metastasis to Bone
Source: Int J Mol Sci. 2021 Jun 27;22(13):6906. doi: 10.3390/ijms22136906 (PMC8269289; doi:10.3390/ijms22136906)
Supplement: Supplementary file 1 [file ijms-22-06906-s001.zip › ijms-1250048-supplementary.pdf]

1. Supplementary Methods

1.1 Tumor Studies

All animal experiments were carried out in compliance with the Vanderbilt University Institutional Animal Care and Use Committee (protocol # M1600176) and the National Institute of Health guidelines. $5x10^5$ MDA-Ctrl or MDA-OEα2 tumor cells were injected into the 4th inguinal mammary fat pad of 4-6 week old athymic nude mice (n=8). After 29 days, primary tumor, peripheral blood, and hindlimbs were collected for downstream analyses.

1.2 Immunohistochemistry.

Immunohistochemistry was performed on 5 μM sections of primary tumor to evaluate the expression of α_2 integrin (1:250, Abcam #ab13355). Briefly, antigen retrieval was performed with citrate buffer followed by blocking in 5% goat serum for 1h and primary antibody incubation overnight at 4^0C. Sections were incubated in an anti-rabbit secondary antibody (Santa cruz, #sc2004) for 2h at room temperature and detected with NovaRed peroxidase kit (Vector Labs). % α2 stained area was evaluated over total tumor area by ROI analysis measurements using Metamorph imaging analysis software. Segmented ROI analysis was also performed to evaluate differential α2 expression along the periphery and center of tumor.

1.3 Flow Cytometry.

Plasma was isolated from the peripheral blood of tumor mice collected via cardiac puncture immediately prior to sacrifice. Bone marrow was isolated from the hindlimbs of mice by centrifugation. Plasma or bone marrow cells were resuspended in red blood cell lysis buffer, incubated for 5 minutes on ice, spun down, and washed twice with PBS. Up to $1x10^6$ cells were stained with 175 ng CD298 antibody (BioLegend, Cat #341704) for 30 minutes on ice in the dark. Samples were run on a 5-laser BD-LSRII in the Vanderbilt Medical Center Flow Cytometry

Shared Resource core and analyzed using FlowJo Software. Whole cells and singlets were gated using forward scatter and side scatter discrimination. CD298+ cells were gated by using a stained sample of pure MDA-MB-231 cells as a positive control.

1.4 Histology

Excised tissues were fixed in formalin for 48 hours, and then stored at 4°C in 70% ethanol. Bones were decalcified in 20% EDTA at 4°C for up to 2 weeks. Tissues were processed, embedded in paraffin, and 5μm thick serial sections were cut on a microtome (Leica Biosystems). Sections were stained with Hematoxylin and Eosin (H&E) to analyze % tumor area. Tumor burden was analyzed in the hindlimbs by freehand ROI analysis in Metamorph and was measured as a percentage of total bone marrow area.

1.5 qPCR

The expression of TGFβrII (Hs00234253_m1) and RUNX2 (Hs01047975_m1) was measured in triplicate by qRT-PCR using validated TaqMan primers from Applied Biosciences (Carlsbad, CA, USA) with the 7500 Real-Time PCR System (Applied Biosystems, Foster City, CA, USA) using the following cycling conditions: 95°C for 15 seconds and 60°C for 1 minute, preceded by an initial incubation period of 95°C for 10 minutes. Quantification was performed using the absolute quantification for human cells method using 18S or GAPDH as an internal control.

1.6 Western Blot Analysis

To measure protein expression changes, cells were harvested in RIPA buffer containing a cocktail of proteases and phosphatase inhibitors (Thermo Scientific, Rockford, USA). Equal protein amount (20 μg) was prepared and run on a 4-20% SDS-PAGE gel. Proteins were transferred to a nitrocellulose membrane and blocked with 5% BSA for 1h at room temperature,

followed by incubation with primary antibodies (TGFβrII: 1:500 Santa Cruz sc-17791, RUNX2: 1:1000 Abcam #ab76956) overnight at 4°C or GAPDH (1:5000 Cell Signaling Technology #2118) loading control. Membranes were then incubated with a secondary antibody at 1:5000 (anti-mouse, Santa Cruz #sc-2005 or anti-rabbit, Santa Cruz #sc-2004) and bands were detected by chemiluminescence using a Chemidoc Touch gel imager (BioRad).

2. Supplementary Figures

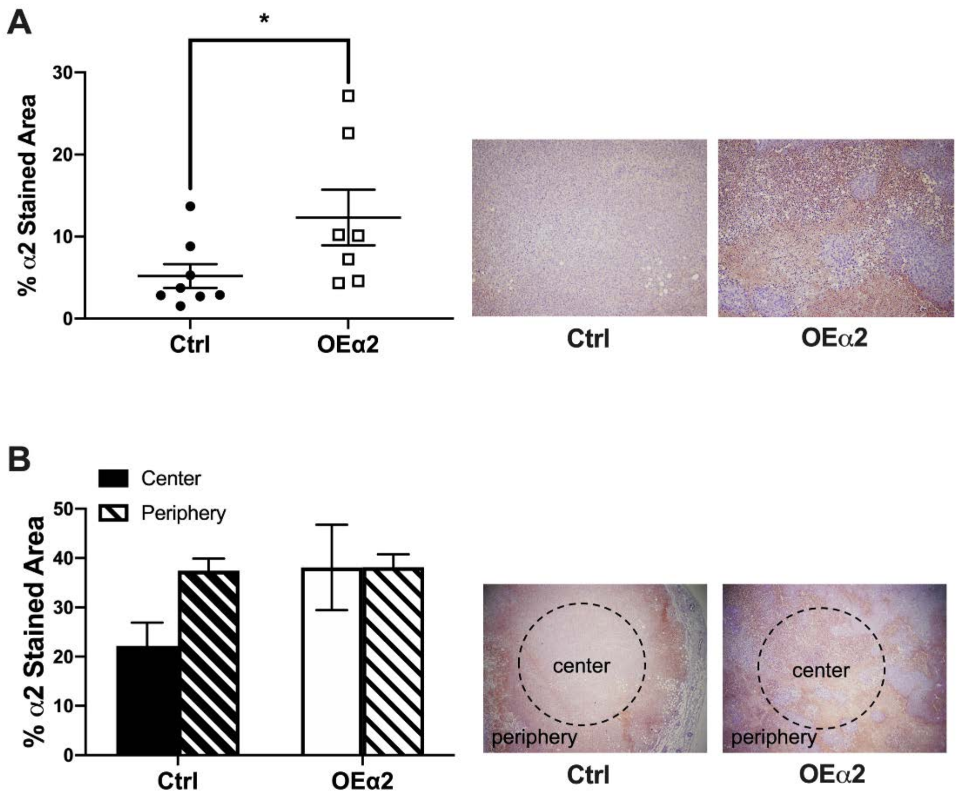

Figure S.1. MDA-OEα2 tumors have high α2 expression. Immunohistochemical staining for α2 integrin subunit confirmed that (**A**) MDA-OEα2 tumors have higher α2 expression than MDA-Ctrl cells. n=8. Mann-Whitney Test, *p<0.05 (**B**) α2 expression in MDA-Ctrl cells is localized more to the periphery of the tumor while MDA-OEα2 cells have more consistent α2 expression throughout the tumor area. Center vs Periphery, 2-way ANOVA, Ctrl p=0.09, OEα2 p=>1. Right

side representative images show Hematoxylin staining, and positive immunohistochemical staining (dark red staining).

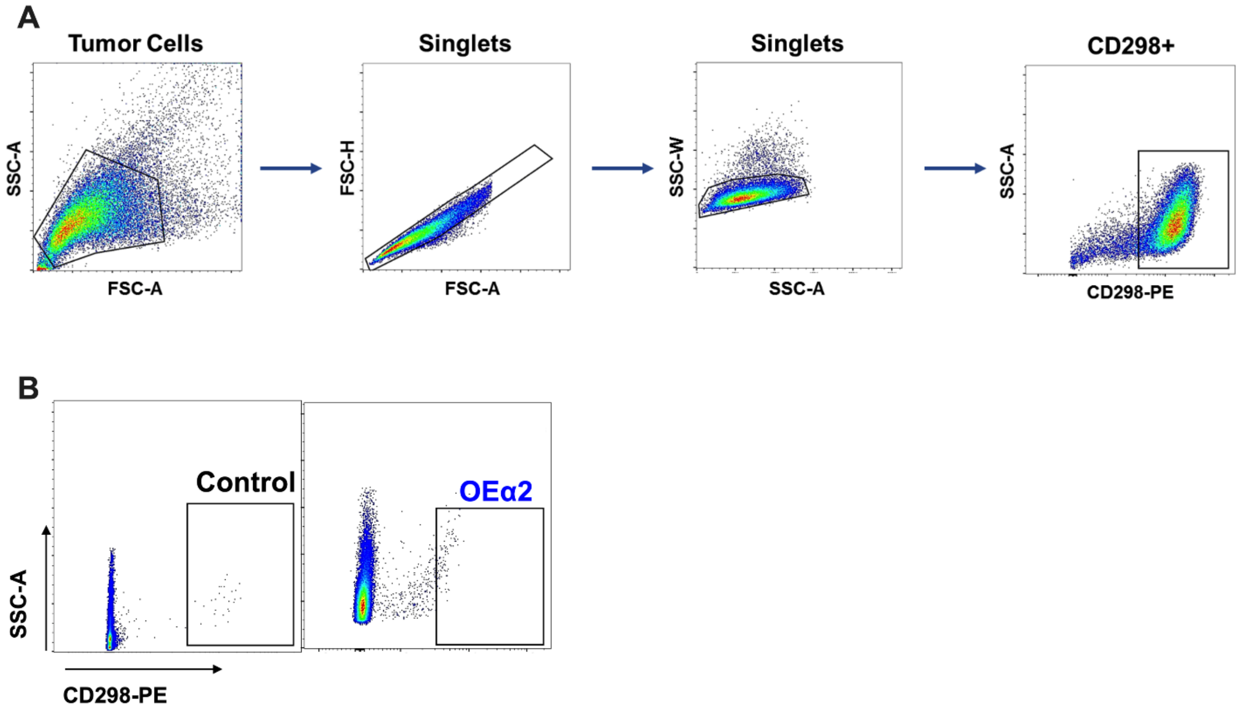

Figure S.2. Flow Cytometry gating scheme for CD298+ tumor cells in mouse bone marrow. (**A**). Whole cells and singlets were gated using forward scatter and side scatter discrimination. CD298+ cells were gated by using a stained sample of pure MDA-MB-231 cells as a positive control. (**B**) Representative flow analysis for CD298+ cells in MDA-Ctrl and MDA-OEα2 samples.

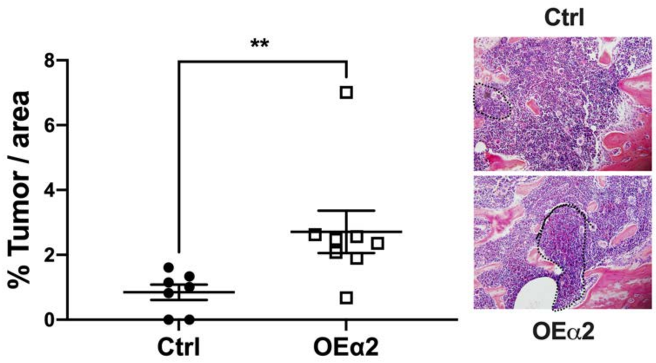

Figure S.3. Quantification of disseminated tumor cells in the hindlimbs of MFP injected mice. H+E staining confirmed the presence of disseminated tumor cells in the bone marrow of tibias from mice injected with MDA-Ctrl or MDA-OEα2 cells via mammary fat pad (MFP) injection. Mice injected with MDA-OEα2 cells had a higher number of disseminated tumor cells compared to mice given MDA-Ctrl cells. Tumor cells are outlined in representative images. n=8. Mann-Whitney Test, ** $p<0.01$

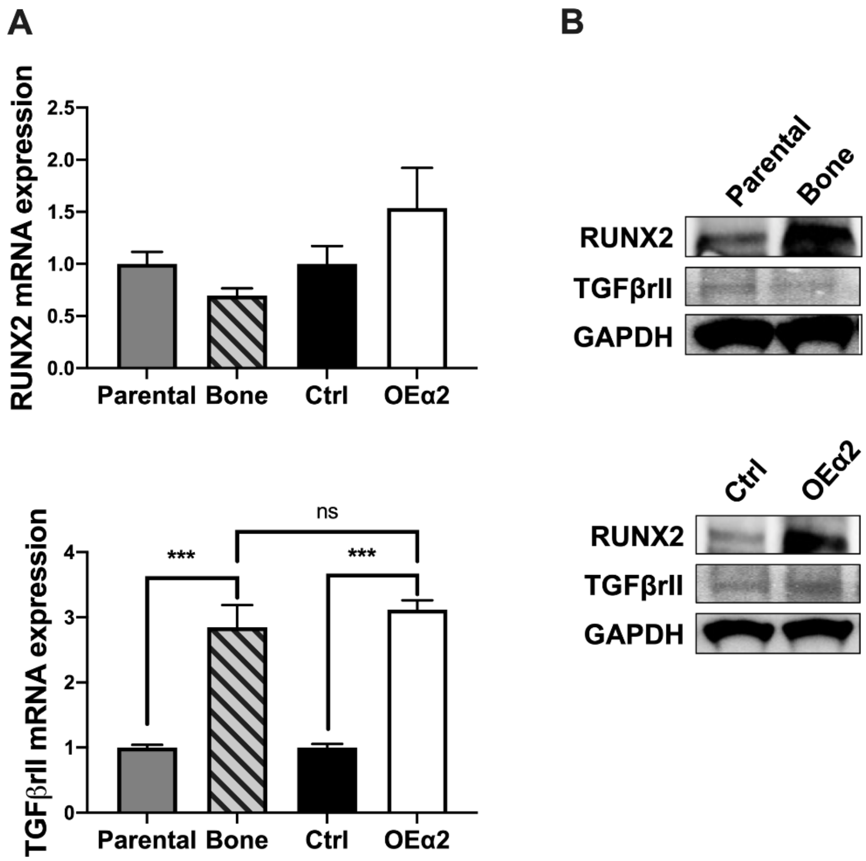

Figure S.4. . Expression of TGFβrII and RUNX2 in OEα2 and bone metastatic MDA-MB-231 cells. MDA-Parental, MDA-Bone, MDA-Ctrl, and MDA-OEα2 cells were analyzed for osteolytic gene expression by (**A**) qPCR and (**B**) Western Blot analysis. No significant change was observed for RUNX2 expression between any of the cell lines, while TGFβrII expression significantly increased in both bone metastatic and OEα2 cells. N=3 biological replicates. One way ANOVA. ***p<0.001.
